# The Domestic Cat as a Large Animal Model for Characterization of Disease and Therapeutic Intervention in Hereditary Retinal Blindness

**DOI:** 10.1155/2011/906943

**Published:** 2011-04-14

**Authors:** Kristina Narfström, Koren Holland Deckman, Marilyn Menotti-Raymond

**Affiliations:** ^1^Department of Veterinary Medicine and Surgery, College of Veterinary Medicine, Mason Eye Institute, University of Missouri-Columbia, MO 65211, USA; ^2^Department of Ophthalmology, Mason Eye Institute, University of Missouri-Columbia, MO 65212-0001, USA; ^3^Department of Chemistry, Gettysburg College, Gettysburg, PA 17325, USA; ^4^Laboratory of Genomic Diversity, National Cancer Institute-Frederick, Frederick, MD 21702-1201, USA

## Abstract

Large mammals, including canids and felids, are affected by spontaneously occurring hereditary retinal diseases with similarities to those of humans. The large mammal models may be used for thorough clinical characterization of disease processes, understanding the effects of specific mutations, elucidation of disease mechanisms, and for development of therapeutic intervention. Two well-characterized feline models are addressed in this paper. The first model is the autosomal recessive, slowly progressive, late-onset, rod-cone degenerative disease caused by a mutation in the *CEP290* gene. The second model addressed in this paper is the autosomal dominant early onset rod cone dysplasia, putatively caused by the mutation found in the *CRX* gene. Therapeutic trials have been performed mainly in the former type including stem cell therapy, retinal transplantation, and development of ocular prosthetics. Domestic cats, having large human-like eyes with comparable spontaneous retinal diseases, are also considered useful for gene replacement therapy, thus functioning as effective model systems for further research.

## 1. Introduction

The value of appropriate animal models to advance our understanding and treatment of human retinal disease processes that cause severe visual impairment or blindness cannot be overemphasized. Animal models have led to the identification of disease genes, and elucidation of the molecular genetic and cellular mechanisms underlying retinal pathology. Moreover, they provide the basis for testing the efficacy of therapeutic approaches, including the use of drugs and gene replacement [1], novel genetic approaches (siRNA) [2], stem cell therapy [3], surgical intervention, such as retinal transplantation [4], and the use of ocular or retinal prosthetics [5]. Additionally, animal models can lead to the identification of novel genes underlying human retinal pathology. Though 157 genes have been identified as causative of nonsyndromic human retinitis pigmentosa (RP; http://www.sph.uth.tmc.edu/retnet/home.htm), over 50% of the genetic causality of RP still remains uncharacterized [6]. 

Though the mouse has been the classic animal model of retinal disease, the advent of comprehensive genetic maps of many mammals has led to the identification of a number of non-rodent animal models of human hereditary retinal disease. Many large animal models offer a complement to existing rodent models. The size of the rodent eye is restrictive for visualization using regular clinical ophthalmic instrumentation and also in conjunction with therapeutic intervention. Even for detailed morphological studies the small size of the mouse eye may be a problem. As a case in point Pazour et al. previously reported that in their research examining the trafficking of ciliary protein in photoreceptor cells, the physical limitations of the mouse retina led them to resort to the use of a bovid eye [7].

## 2. Dogs and Cats as Large Animal Models of Spontaneous Retinal Disease

Dog and cat populations offer a wealth of potential as large animal models of human retinal disease. Small effective population sizes, the use of popular sires and inbreeding have contributed to the “load” of inherited diseases, especially in dog breeds [8]. Hereditary and primary photoreceptor diseases, or progressive retinal atrophies (PRA) have been described in more than 100 dog breeds [9], many of which are likely to be caused by the same mutation which is observed across related breeds. This phenomenon has been observed in a number of gene-defining phenotypes in the dog [10–12]. Thirteen genes have been mapped and characterized as causative of canine PRA, including *ADAM9* [13],* CCDC66 *[14],* CNGB3* [15], *PDE6*α** [16], *PDE6*β** [17]*, PRCD* [18], *RD3* [19], *RHO* [20], *RPE65* [21], *RPHP4* [22], *RPGR* [23], *RPGRIP1* [24], and *VMD2* [25]. 

Cats have been considered to be affected less frequently by hereditary disease. However, the informative website Online Mendelian Inheritance in Animals (http://omia.angis.org.au/) catalogues 288 distinctive pathologies with an inherited component in the cat, with cited references. Only in recent years have specific mutations been elucidated for hereditary retinal diseases in cats [26, 27], clinically similar to the PRA complex in dogs [28]. Domestic dogs and cats of today experience a level of medical surveillance second only to human kind, thus increasing the likelihood, that individuals with rare or unique mutations are identified.

## 3. Sequencing of the Cat Genome

Report of two partial sequences (1.9X, 3X) of the cat genome [29, 30] has been invaluable in the initial mapping and characterization of feline hereditary diseases [26, 27, 31]. A full genome sequence (14X) of the cat has currently been completed (Wes Warren, Washington University, personal communication). The identification of single-nucleotide polymorphisms (SNP) in cat breeds, an integral part of the 14X full genome sequence project and the 3X sequencing of the cat genome [30] is currently being utilized in development of a domestic cat SNP chip. With the availability of these genomic resources, the mapping and characterization of feline monogenic disorders will largely be dependent on obtaining an appropriate sample set. Genome-wide association (GWA) studies in dog breeds are proving extremely successful in identifying genes associated with breed-defining phenotypes and monogenic disorders [13, 32, 33]. Often this is accomplished with surprisingly small sample sizes. The mapping of the canine cone-rod dystrophy 3 gene (*ADAMS9*) in the Glen of Imaal Terrier breed was recently accomplished with as few as 22 unaffected and 19 affected individuals [13] while Awano et al. reported identification of the gene causative of canine degenerative myelopathy (*SOD1*) in the Pembroke Welsh corgi with 38 affected and 17 control individuals. Extended linkage disequilibrium (LD) in dog breeds [19, 34, 35] contributes to the success of GWA mapping in dogs and is an important factor underlying successful mapping with small sample sizes in the dog. Preliminary studies suggest that extended blocks of LD are also observed in cat breeds, though the length of LD appears to be abbreviated to that which is observed in dog breeds [29].

## 4. The Abyssinian Retinal Degeneration Cat Model (*rdAc*)

**Figure 1:**
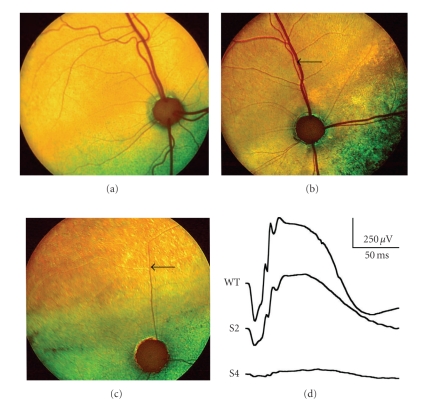
Fundus appearance and electroretinograms (ERGs) of *rdAc *individuals with the *CEP290* mutation. Fundus photographs demonstrate (a) a 1-year-old unaffected Abyssinian cat (wildtype, WT), (b) a 2-year-old affected Abyssinian cat with an early disease stage (S2) [39], and (c) a 6-year-old Abyssinian with an advanced disease stage (S4) [39]. Arrows in (b) and (c) indicate retinal vasculature that is attenuated, more so in the advanced stage (c) than in early stage of disease (b). For the same three cats, the waveforms of the dark-adapted full-field flash ERG recordings are shown, using 4 cd.s/m^2^ of white light stimulation for each of the recordings. Amplitude and implicit time calibrations are indicated in the figure, vertically and horizontally, respectively. Reproduced with permission from [43].

**Figure 2:**
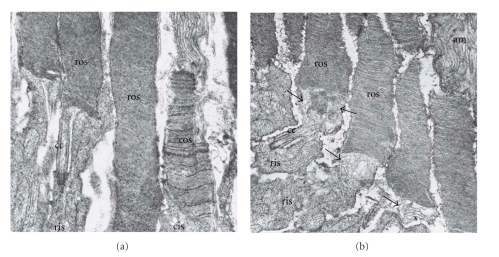
Electron micrographs of outer retina showing photoreceptor outer and inner segments of normal (a) Abyssinian cat and young affected (b) *rdAc* cat. Note abnormalities at the base of the rod outer segments near the connecting cilium in (b); membranes are not formed as in the normal (a), instead there is vacuolization and degeneration (arrows) of membranes in the affected retina. Am: apical microvilli of the retinal pigment epithelium, ros: rod outer segments, ris: rod inner segments, cos: cone outer segments, cis: cone inner segments, cc: connecting cilium of the photoreceptor. Original magnification: ×19152. Reproduced with permission from [27].

The female Abyssinian cat (Cinnamon), subject of the feline whole genome sequencing efforts, is a member of a pedigree developed for genetic mapping of the gene defect for the *rdAc (retinal degeneration in Abyssinian cats) *model, first described in 1982 [36]. The autosomal recessive (AR) trait, *rdAc, *has become an important model of human RP [37, 38]. At birth, affected cats have normal vision, but, by 1.5–2 years of age they develop early changes that can be observed by ophthalmoscopy [39] (Figures 1(a)–1(c)). By 7 months of age, affected cats demonstrate significantly reduced retinal function by electroretinography (ERG; Figure 1(d)). ERG a-wave amplitudes are then reduced more than 50% as compared to normal individuals, with a parallel reduction in retinal oxygen tension [40]. Rod photoreceptor outer segments exhibit the first morphological changes in individuals 5–8 months of age, observed as a disorganization and disruption of rod outer segment lamellar discs and the appearance of vacuoles near the connecting cilium [41]. Progression of the disease results in further degeneration of the rods (Figures 2(a) and 2(b)), followed also by disruption of the cone photoreceptors. By 3–5 years of age, the clinical end stage of the disease has been reached with generalized photoreceptor degeneration, and subsequently retinal atrophy leads to blindness [42].

**Figure 3:**
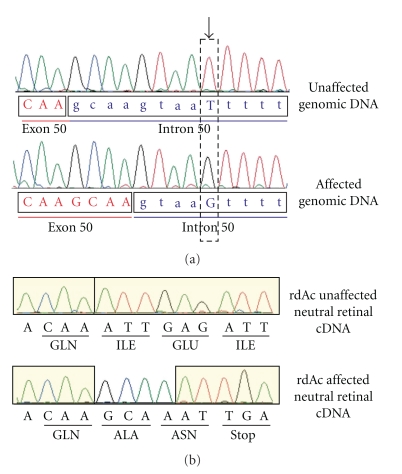
(a) Electropherograms of genomic DNA of *CEP290* sequenced in unaffected and *rdAc* affected individuals of exon 50/intron 50 junction. Arrow indicates position of SNP in intron 50, which uncovers a canonical GT splice donor site, resulting in alternative splicing in affected individuals. Exon 50 (red letters) and intron 50 (blue letters) nucleotides were identified by cDNA sequence analysis. GenBank Accession No. for feline *CEP290*: EF028068. (b) Electropherograms of cDNA for *CEP290* 3′ region of exon 50 generated from neural retinal tissue in affected and unaffected individuals. Alternative splicing in affected individuals results in a frame shift and introduction of a premature STOP codon. Reproduced with permission from [27].

The molecular genetic basis of* rdAc* was recently established in the *CEP290 *gene. A single-nucleotide polymorphism in an intron of the felid *CEP290 *gene generates a novel strong canonical splice-donor site resulting in a 4-bp insertion, a frame shift, and the introduction of a premature stop codon (Figures 3(a) and 3(b)). The putative truncated CEP290 peptide would lack the more 3′ KIDV and VI domains. The protein is an important component of the intraflagellar transport (IFT) system whereby specialized proteins critical for phototransduction are transferred from their site of synthesis in the inner segment of photoreceptors through the connecting cilium to the outer segment [7]. As the rod photoreceptor discs are in a constant state of regeneration, a fully functional IFT system is critical for the maintenance of the photoreceptors [7]. In the *rd16* mouse model, the phototransduction proteins opsin and rhodopsin are found concentrated in the inner segment, which led Chang et al. [44] to propose a ciliary trafficking role for the CEP290 protein. Mutations in *CEP290* have been reported in RP, Leber's congenital amaurosis (LCA), as well as the syndromic retinopathies, Joubert, Meckel-Gruber, and Bardet-Biedl [45–48].

Recently, it has been shown that *rdAc* cats exhibit some degree of phenotypic variation, with end-stage blindness reached in individuals anywhere from three to seven years [49]. Interestingly, it appears that the slow progression of disease may be one of the factors leading to the cat's exceptional ability to adapt to decreasing retinal function [49]. The condition thus evaded detection by both owners and veterinarians in a highly popular cat breed, the Siamese, which demonstrates a high frequency (*∼*33%) for the *rdAc *disease allele [49]. Breeding practices have caused the *CEP290* mutation to spread to multiple cat breeds [49], and to exhibit a worldwide distribution [49].

## 5. The Rod Cone Dysplasia Cat Model (*Rdy*)

A second feline model of human retinal dystrophy, the *Rdy* cat, was first described in a single Abyssinian cat, from which a pedigree was developed and extensively studied on a phenotypic level [50–53]. The disease is an early onset primary photoreceptor disorder with an autosomal dominant (AD) mode of inheritance in which affected individuals exhibit abnormal photoreceptor development at 22 days of age. The disease leads rapidly to blindness usually within the first few months of life. Further characterization of the dystrophy has demonstrated that the photoreceptors never develop normally, and the disease has therefore been designated as a rod-cone dysplasia with early onset degeneration of both cones and rods. 

**Figure 4:**
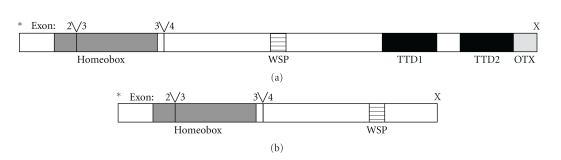
CRX protein structure in *Felis catus*. Wildtype feline CRX protein (a) compared to the putative truncated CRX protein (b). The exon splice junctions are noted as “Y”. The start codon and stop codons are labeled as (*) and (X), respectively. The protein domains are highlighted as shaded boxes and defined as the homeobox, the WSP domain, the transcriptional transactivation domains 1 and 2 (TTD1 and TTD2), and the OTX tail. Domains are drawn to scale. Reproduced with permission from [26].

The molecular genetic basis for *Rdy* was recently elucidated [26]. A single-base deletion in the *CRX* gene introduces a frameshift and a stop codon immediately downstream, truncating a region previously demonstrated as critical for gene function [26, 54] (Figure 4). The *CRX* gene product is critical in transcriptional activation of a number of genes involved in photoreceptor development and maintenance [55, 56]. In humans, mutations in *CRX* are associated with human AD cone-rod dystrophy (CoRD), and both AD and AR Leber's congenital amaurosis (LCA) [54, 57–59]. The *Rdy* cat is the first large animal model for* CRX*-linked spontaneous retinal disease. A large screening of cat breeds has failed to detect any other domestic feline breeds with the disease allele [26]. 

The *Rdy *model provides one of the very few large animal models of an autosomal dominant retinal disease. These disorders are challenging from a therapeutic standpoint. Causality of the disease can arise from haploinsufficiency of product, or in some circumstances from gain of function or competition from a truncated or aberrant protein product [60]. The presence of both mutant and wildtype RNA in *Rdy* individuals, initially suggestive that a truncated CRX product might be generated [26] has been supported by recent findings (K. Holland Deckman, unpublished). The truncated peptide would retain the CRX motifs involved in nuclear localization and DNA-binding, but lack the region critical for transcriptional activation of photoreceptor specific genes [61]. This truncated product could thus compete with the wildtype CRX product and other transcription factors for promoter binding regions of target genes, which is currently under investigation.

## 6. Other Cat Models under Investigation

In the late 1960s, a new feline breed, the Bengal, which has gained huge popularity, was developed through hybridization of domestic cats and the Asian leopard cat [62]. Recently, a novel, early onset autosomal recessive disorder was described in this breed [63]. The disease is under investigation but appears to be an early onset primary photoreceptor disorder, leading to blindness within the first year of age. Genetic mapping and further characterization of the disorder are in progress. A second feline retinal disease model has been described in the Persian cat breed [64]. The rod cone dysplasia demonstrates an autosomal recessive mode of inheritance [64]. Affected individuals showed clinical signs of disease 2-3 weeks after birth and clinical blindness at 16 weeks of age. Photoreceptors in affected individuals never reach full maturity. The molecular genetic defect for both of these disorders has not as yet been elucidated. It appears that the Bengal cat retinal disease should become an important animal model for the research community in regards to the study of various treatment modalities. The disease starts out from a mainly normal appearing retina but due to the fast progression of the disorder, retinal atrophy ensues comparatively early thus functioning as an effective model system for retinal research.

## 7. Therapeutic Intervention

The retinas of large animal models more closely approximate that of humans, and are thus more easily amenable for visualization and imaging [65] of the disease process, for surgical intervention, and for clinical evaluation of therapeutic effects. Dogs and cats also offer the potential of long-term followup studies in conjunction with treatment trials. 

The rod and cone photoreceptors (the latter; short and middle wavelength sensitive cones) of both species are distributed in the retina in a mosaic pattern comparable to that of the human retina. Neither cats nor dogs have a macula. However, in cats, in the same region as the human macula, there sre a cone-rich region called the area centralis where the concentration of cones in comparison to that of rods is higher than at any other location. Along with the holangiotic configuration of the retinal vasculature, the cat retina becomes structurally similar to the human counterpart. Further, cats in particular, have historically been important models in neuroanatomy and neurophysiology, especially with respect to visual function. 

Successful therapeutic intervention is the ultimate goal of research using animal models for human retinal disease processes. In recent years, groundbreaking research has been performed by independent groups in regards to gene therapy using dogs with spontaneous hereditary retinal disease. Proof of principle that the technology works was achieved by an *in vivo* study by Acland et al. [66], using AAV2/2 as a safe and effective vector. The well-characterized *rdAc* and* Rdy* feline models of spontaneous hereditary retinal disease, now with known mutations, are excellent candidates for gene therapy-based approaches, especially for the late onset type of retinal degeneration (Jean Bennett, personal communication, 2007). Gene therapy approaches targeting the *Rdy* model, which has been recently elucidated on the molecular genetic level, are currently under investigation. 

Novel therapeutic interventions have recently been developed to target aberrant RNA species that survive nonsense mediated decay. Short interfering RNA (shRNA), short double stranded RNA molecules, can be designed to degrade specific target mRNAs [67], while ribozymes, which are small catalytic RNAs, are designed to cleave complementary RNA sequences [2]. RNA interference-mediated suppression and replacement aims to remove both wildtype and aberrant RNA copies of a targeted gene while replacing wildtype expression with a copy of the gene. 

Other methods of treatment include retinal transplantation of viable cells or tissue. Experimentation in this regard includes the replacement of dying visual cells with healthy neuroblastic progenitor cells and retinal pigment epithelial (RPE) cells as sheets of normal tissue [68]. It has been demonstrated that retinal transplants in rats can morphologically reconstruct a damaged retina and restore retinal sensitivity [69]. Affected cats with the *CEP290 *defect (*rdAc*) have been used in trials with transplantation of sheets of allogeneic fetal retinal tissue [70]. Surgeries have been successful as to graft survival in the retina, although cellular connectivity has not been shown and ERG testing has not demonstrated improvement in retinal function. So far the cat model in regards to transplantation of large sheets of normal tissue has shown a comparatively high risk for complications. The tight structures of the cat eye presents difficulties to manipulate the globe in the orbit in comparison to other large animal models (such as dog, pig, and rabbits) and the high frequency of hemorrhage from the deep venous plexus region of the domestic cat renders this surgery difficult even for experienced surgeons [71]. 

Transplantation of stem and neural progenitor cells appears to offer considerable promise. Subretinal transplantation of neural progenitor cells in rats has shown evidence of cellular repopulation of damaged retinas and retardation of ongoing retinal degeneration [72, 73]. Neural progenitor cells can also be engineered to secrete specific growth factors such as glial cell line-derived neurotrophic factor (GDNF). When used for transplantation studies such cells contributed to enhanced cellular survival, neuronal differentiation, and improved host cognitive function following brain injury, in comparison to transplantation of nontransduced neuronal progenitor cells [74]. Recent studies, using *rdAc* animals have shown promising results when retinal progenitor cells from transgenic fluorescent red cats were transplanted to cats affected by the *CEP290* mutation (*rdAc*) by subretinal injections of progenitor cell suspensions. No adverse reactions were observed in the transplanted cat eyes. There was further development and migration of transplanted cells in the outer and inner retina, and development of donor progenitor cells specifically into Müller-like cells observed by immunohistochemistry [63]. Further studies are in progress. 

Another modality under development using the feline species is intraocular implantation of retinal prosthesis [75]. Either epiretinal or subretinal implantation can be utilized in the degenerate retina. The electrodes in the prosthesis may emit electrical currents and stimulate residual retinal cells, such as second- and third- order neurons, for example, bipolar and ganglion cells. Signals to the visual cortex are transmitted to produce a visual sensation. It appears that the cat eye, with the visual processes already thoroughly investigated, would be an optimal animal model for further development of research in regards to retinal prosthesis.

## 8. Future Directions

Through discoveries of causative mutations and their detrimental effects on retinal cell function, new insights into retinal degenerative disease mechanisms have been gained. It is now possible to aim therapies at correcting disease mutations in the eye directly or indirectly. The cat species, with disease entities that are comparable to those of humans, and with large human-like eyes, is amenable to treatment using similar surgical techniques and instrumentation as those used for humans. We now have an effective model system that can be used for cell replacement therapy, retinal transplantation using tissue from healthy retinas or retinal progenitor cells, artificial retinal prosthesis, or combinations of one or more of the above. There definitely is some hope of further advancement in the field of spontaneously occurring hereditary retinal blinding disease using the cat as a valuable large animal model.
